# Feasibility of Diffusion Tensor Imaging at 1.5T Using Multi-Band Echo Planar Acquisition

**DOI:** 10.2463/mrms.tn.2015-0159

**Published:** 2016-09-06

**Authors:** Minoru Mitsuda, Yuichi Suzuki, Akira Kunimatsu, Akihiro Kasahara, Yasushi Watanabe, Kenji Ino, Keiichi Yano, Kuni Ohtomo

**Affiliations:** 1 Department of Radiological Technology, The University of Tokyo Hospital, 7-3-1, Hongo, Bunkyo-ku, Tokyo 113-8655, Japan; 2 Department of Radiology, The University of Tokyo Hospital, Tokyo, Japan

**Keywords:** diffusion tensor imaging, parallel imaging, multi-band, simultaneous multislice imaging, tractography

## Abstract

We report that diffusion tensor imaging (DTI) and tractography (DTT) of the pyramidal tracts using multi-band (MB) EPI could be a useful tool with a 1.5T MRI. We compared images using single-band EPI (SB-EPI) and MB-EPI. MB-EPI could reduce the scanning time by about 40%. We demonstrated that it is comparable between image qualities of SB-EPI and MB-EPI using tract-specific analysis and dice coefficients. Therefore, MB-EPI can promote high-speed DTI and DTT in clinical applications.

## Introduction

Recently, microstructure analysis of the brain (structural, functional, diffusion, etc.) by means of magnetic resonance imaging (MRI) has been actively investigated.^1^ In such analyses, high-resolution isotropic images are very useful. However, numerous slices are necessary to cover the whole brain, which poses a major drawback in that the imaging time is increased. To address this problem, simultaneous imaging of multiple slices is useful.

The simultaneous imaging technique was originally demonstrated by Larkman et al. using a gradient-recalled echo sequence.^2^ Those authors reported that simultaneous excited slices could be separated by means of coil-sensitivity profiles. However, it is difficult to separate the simultaneous excited slices when coil-sensitivity profiles are similar among these slices. In the multi-band (MB) technique, several slices are concurrently excited using a single radio-frequency (RF) pulse tailored for selective excitation at multiple frequencies. These slices are then subsequently separated based on the coil-sensitivity profiles and the phase-shift technique, which uses the phase of the RF pulses to shift the position of adjacent slices in such a way as to reduce overlap between aliased slices in the image space. This facilitates separation of aliased slices where the coil-sensitivity profiles are similar.^3–5^

The MB technique was applied to the echo planar imaging (EPI) sequence in a number of studies.^5–7^ MB can acquire more high-resolution images by increasing the number of slices without extending the repetition time (TR) and acquisition time, and can reduce acquisition time while maintaining image resolution.

MRI using diffusion phenomena is useful for various analyses, including the commonly used diffusion-weighted imaging (DWI), q-space imaging (QSI), and diffusion spectrum imaging (DSI).^8,9^ As other examples of diffusion MRI, diffusion tensor imaging (DTI) and diffusion tensor tractography (DTT) have been proposed as noninvasive techniques for identifying white matter tracts *in vivo*, and the clinical utility of these techniques has been already reported.^10–16^

There are, however, no reports quantitatively evaluating DTI and DTT using MB-EPI. Furthermore, most previous reports on the MB sequence have used high-spec devices (e.g., high field [≥ 3T] and multi-channel coils [≥ 32-channels]). In this study, we demonstrate that DTI and DTT using MB-EPI could be a useful tool even with a 1.5T MRI, which is commonly used clinically.

## Materials and Methods

### MR imaging

Subjects were 10 male healthy volunteers (mean age: 26.6 years). This study was approved by the institutional ethics committee of the University of Tokyo, and only subjects who gave written informed consent were included in the study.

All MR images were acquired using a 1.5T clinical scanner (MAGNETOM Avanto; Siemens Healthcare, Erlangen, Germany; 45 mT/m maximum gradient strength, 200 mT·m^−1^·s^−1^ maximum slew rate) and a commercial 12-channel matrix head coil. The MB-EPI sequence used in this study was Release R011a for VB17A (https://www.cmrr.umn.edu/multiband/#refs).

The imaging parameters for conventional single-band EPI (SB-EPI) were TR / echo time (TE) = 8900 / 88 [ms]; in-plane acceleration factor (generalized autocalibrating partially parallel acquisitions [GRAPPA] factor) = 2; slice thickness / gap = 2.5 / 0 [mm]; number of slices = 60; field of view (FOV) = 245 × 245 [mm^2^]; matrix size = 98 × 98; voxel size = 2.5 × 2.5 × 2.5 [mm^3^]; b = 1000 [s/mm^2^]; 30 directions; and single b = 0 [s/mm^2^] image. The imaging parameter for MB-EPI were TR / TE = 4200 / 73.4–83.8 [ms]; GRAPPA factor = 2; slice acceleration factor (MB factor: MBf) = 2–4; slice thickness / gap = 2.5 / 0 [mm]; number of slices = 60; FOV = 245 × 245 [mm^2^], matrix size = 98 × 98; voxel size = 2.5 × 2.5 × 2.5 [mm^3^]; b = 1000 [s/mm^2^]; 30 directions; and single b = 0 [s/mm^2^] image. The acquisition times were 4 min 30 s for SB-EPI, 2 min 49 s for MB-EPI using MBf2, 2 min 37 s for MB-EPI using MBf3, and 2 min 42 s for MB-EPI using MBf4. In addition, 3-dimensional T_1_-weighted images (MP-RAGE, TR / inversion time / TE = 1700 / 800 /3.3 [ms]; flip angle = 15 [degree]; GRAPPA factor = 2; slice thickness / gap = 1.25 / 0 [mm]; number of slabs = 120, FOV = 288 × 288 [mm^2^]; matrix size = 192 × 192; voxel size = 1.5 × 1.5 × 1.25 [mm^3^]; acquisition time = 4 min 49 s) were acquired for anatomical information.

### Data processing

The distortion caused by eddy currents was corrected for all DWI data using a software from the Oxford Centre for Functional MRI of the Brain (FSL v.5.0.7; FMRIB Software Library, http://www.fmrib.ox.ac.uk/fsl/).^17^ b0-images and T_1_-weighted images were coregistered using MATLAB2012b (Mathworks; Natick, NA) and Statistical Parametric Mapping 8 (SPM8, www.fil.ion.ucl.ac.uk/spm/software/spm8/ ), and coregistered T_1_-weighted images were eliminated, except for the cerebral parenchyma, using MRIcron ( http://www.mccauslandcenter.sc.edu/mricro/mricro/index.html). Diffusion toolkit was used for tensor analysis of corrected DWI.^18^ The parameters on diffusion toolkit were set the initial value. The target region of interest (ROI) was set at the primary motor cortex on T_1_-weighted images, using VOLUME-ONE and dTV II.FZR (developed by Yoshitaka Masutani; Hiroshima City University). Seed ROIs were set at the cerebral peduncle on a fractional anisotropy (FA) map, using TrackVis.^18^ Tractography of the pyramidal tracts was performed using these ROIs and TrackVis. The Interpolated Streamline method was used for the tractography algorithm.^19^

### Image analysis

We evaluated the DTT similarity of SB-EPI and MB-EPI using the Dice similarity coefficients (DSC). The DSC was calculated using the following equation:

$$\text{DSC}=\frac{2\times V\left(X\cap Y\right)}{V(X)+V(Y)}$$

where X represents a voxel of DTT from SB-EPI data, Y represents a voxel of DTT from MB-EPI data, and V represents the volume of the relevant voxel. DSC < 0.40, 0.40 ≤ DSC < 0.60, 0.60 ≤ DSC < 0.75, and 0.75 ≤ DSC indicate poor, fair, good, and excellent similarity, respectively.^20^

Next, we implemented tract-specific analysis (TSA) to evaluate the apparent diffusion coefficient (ADC) and FA, to compare each MBf of MB-EPI with SB-EPI.^21^ ROIs of TSA were set at the both right and left pyramidal tracts on DTT of SB-EPI.

### Statistical analysis

We compared ADCs and FAs from TSA, for each MBf of MB-EPI and SB-EPI using Dunnett’s test implemented in commercially available software (SPSS v.20; IBM, Tokyo, Japan). *P* < 0.01 was considered significant.

## Results

**Fig 1.**
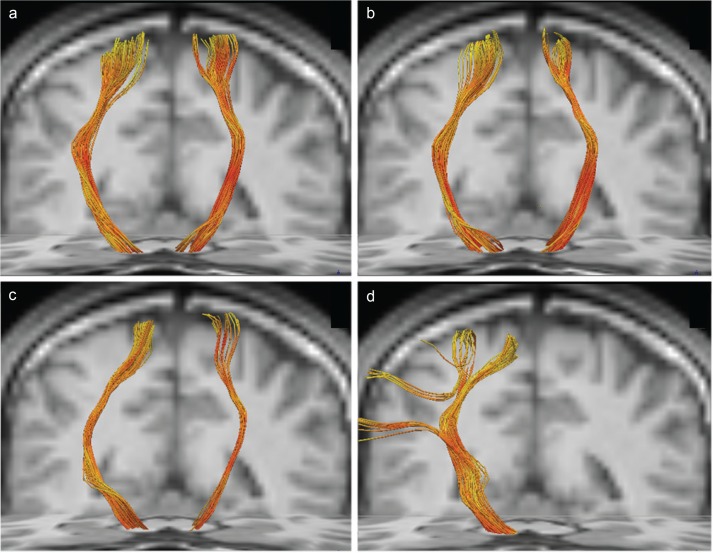
Diffusion tensor tractography (DTT) of pyramidal tracts on T_1_-weighted imaging under the following conditions: SB-EPI (**a**), MBf2 (**b**), MBf3 (**c**), and MBf4 (**d**). As MBf increased, DTT of the pyramidal tracts was less accurately imaged than with SB-EPI, and MBf4 resulted in mistracking.

**Fig 2.**
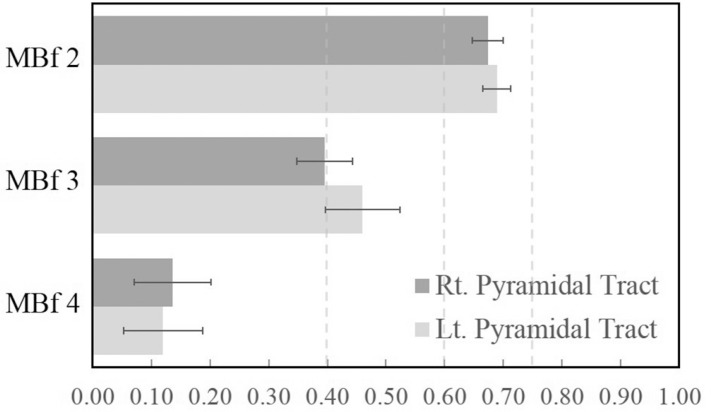
The bars show the DSC (mean ± standard error) in diffusion tensor tractography (DTT) of MBf2 (top), MBf3 (middle), and MBf4 (bottom). DSC indicates the similarity of DTT with SB-EPI. A DSC < 0.40, 0.40 ≤ DSC < 0.60, 0.60 ≤ DSC < 0.75 and 0.75 ≤ DSC indicate poor, fair, good, and excellent similarity, respectively.

Using MB-EPI, scan time was reduced by approximately 40% as compared with SB-EPI. The DTT of each condition is shown in Fig. 1. The imaging capacity of DTT using MBf3 and MBf4 was markedly lower than that using SB-EPI. DSC were 0.67 ± 0.03 (average ± standard error [SE]) and 0.69 ± 0.02 in the right- and left-pyramidal tracts of MBf2; 0.40 ± 0.05 and 0.46 ± 0.06 in right- and left-pyramidal tracts of MBf3; and 0.14 ± 0.07, 0.12 ± 0.07 in the right- and left-pyramidal tracts of MBf4, respectively. The DSC was considered good in MBf2, fair in MBf3, and poor in MBf4 (Fig. 2).

**Fig 3.**
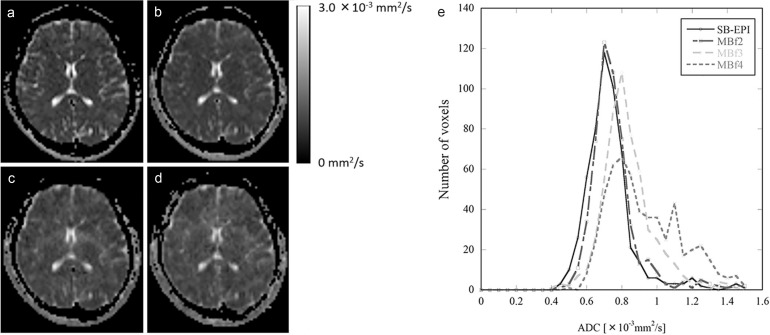
Apparent diffusion coefficient (ADC) maps for each of the following conditions: SB-EPI (**a**), MBf2 (**b**), MBf3 (**c**), and MBf4 (**d**). Note that the ADC map in (**c**, **d**) appears brighter than that in (**a**, **b**). The corresponding voxel histogram of ADC maps (**e**). SB-EPI and MBf2 were very similar. The histograms of MBf3 and MBf4 show the distribution with higher peak.

**Fig 4.**
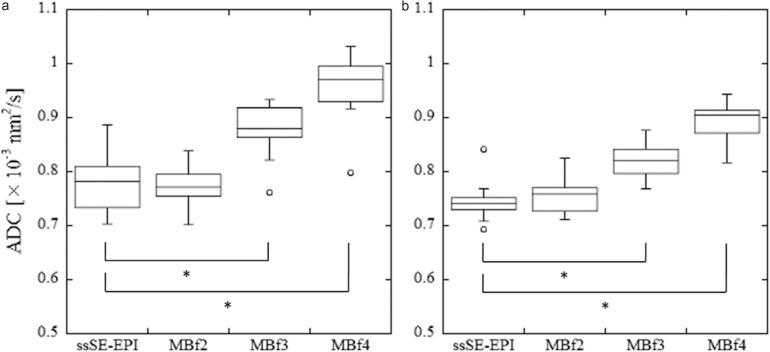
These box plots show the results of tract-specific analysis (TSA) for apparent diffusion coefficients (ADC) of SB-EPI, MBf2, MBf3, and MBf4 under four different conditions: right- (**a**) and left-pyramidal tracts (**b**). Asterisks indicate significant difference (*P* < 0.01) compared to SB-EPI.

In the TSA evaluation, the average ± standard deviation (SD) of ADCs were 0.78 ± 0.06 (×10^−3^ mm^2^/s) and 0.75 ± 0.04 (×10^−3^ mm^2^/s) in the right- and left-pyramidal tracts of SB-EPI; 0.77 ± 0.04 (×10^−3^ mm^2^/s) and 0.76 ± 0.04 (×10^−3^ mm^2^/s) in the right- and left-pyramidal tracts of MBf2; 0.88 ± 0.05 (×10^−3^ mm^2^/s) and 0.82 ± 0.04 (×10^−3^ mm^2^/s) in the right- and left-pyramidal tracts of MBf3; and 0.96 ± 0.07 (×10^−3^ mm^2^/s) and 0.90 ± 0.04 (×10^−3^ mm^2^/s) in the right- and left-pyramidal tracts of MBf4, respectively. The ADCs of MBf3 and MBf4 were significantly higher than that of SB-EPI (Figs. 3, 4).

**Fig 5.**
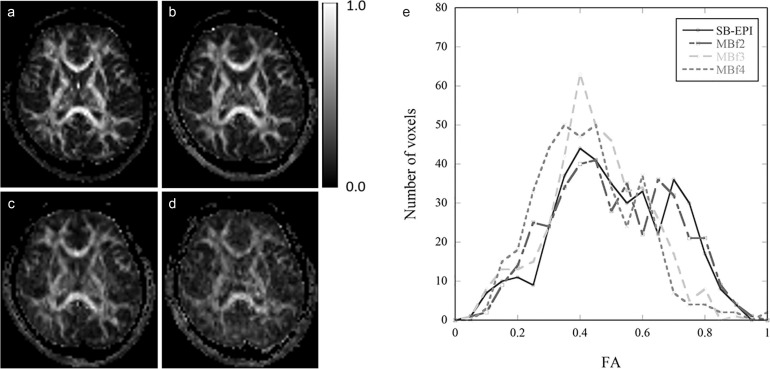
Fractional anisotropy (FA) maps for each of the following conditions: SB-EPI (**a**), MBf2 (**b**), MBf3 (**c**), and MBf4 (**d**). Note that the FA map in (**c**, **d**) appears darker than that in (**a**, **b**). The corresponding voxel histogram of FA maps (**e**). SB-EPI and MBf2 were very similar. The histograms of MBf3 and MBf4 show different distribution in comparison with SB-EPI.

**Fig 6.**
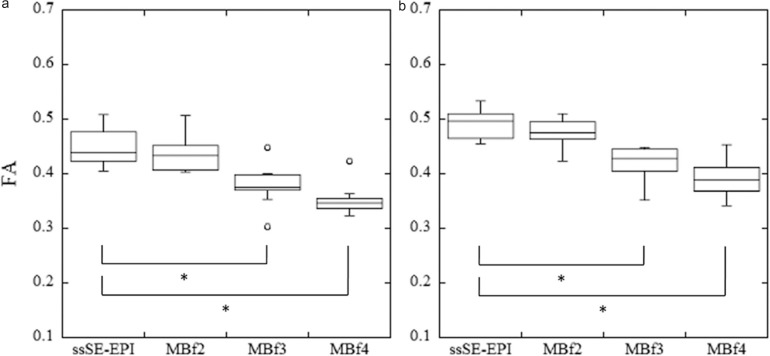
These box plots show the results of tract-specific analysis (TSA) for fractional anisotropy (FA) on SB-EPI, MBf2, MBf3, and MBf4 under four different conditions: right- (**a**) and left-pyramidal tracts (**b**). Asterisks indicate significant differences (*P* < 0.01) as compared to SB-EPI.

The average ± SD of FAs were 0.45 ± 0.03 and 0.49 ± 0.03 in the right- and left-pyramidal tracts for SB-EPI; 0.44 ± 0.03 and 0.47 ± 0.03 in the right- and left-pyramidal tracts for MBf2; 0.38 ± 0.04 and 0.42 ± 0.03 in the right- and left-pyramidal tracts for MBf3; and 0.35 ± 0.03 and 0.39 ± 0.03 in the right- and left-pyramidal tracts for MBf4. The FA for MBf3 and MBf4 were significantly lower than that for SB-EPI (Figs. 5, 6).

## Discussion

Using MBf2 of MB-EPI could reduce the scan time by approximately 40% as compared with SB-EPI. In this study, we used fixed TR (4200 ms) in MB-EPI. Higher MBf could use a shorter TR (e.g., minimum TR = 2500 ms in MBf3), but it is not practical because of T_1_ saturation effects. It seems that the amount of reduction in scan time that can be gained by using MB-EPI is limited. Additionally, MB-EPI (SE-type) has other limitations, due to the peak power of the RF pulse and/or specific absorption rate. Recently, however, it has been reported that these drawbacks are beginning to be resolved,^22,23^ and further improvement is to be expected.

During evaluation of TSA, the ADC and FA of MBf2 was comparable to SB-EPI. The DSC of MBf2 was also comparable to that found in a previous study (DSC = ca. 0.7) that investigated the repeatability of DTT.^20^ We believe that MB-EPI using MBf2 is superior to SB-EPI in terms of the balance between scan time and image quality, because MB-EPI using MBf2 can reduce scan time and maintain imaging quality. More specifically, in terms of clinical utility, MB-EPI is particularly useful for patients who cannot maintain the same posture for an extended period.

On the other hand, the ADCs and FAs were significantly different, and the DSC were lower, in MBf3 and 4. The leakage factor, which is a factor related to residual aliasing among simultaneously excited and acquired slices, increases when MBf is set higher, and this results in a reduction in image quality.^6^ In preliminary phantom examination, the relative leakage factors to SB-EPI have increased by a factor of 1.1 in MBf2, 2.3 in MBf3 and 3.2 in MBf4 (data not shown). Thus, it appeared that the results of MBf3 and 4 are caused by an increased leakage factor in our study. Recently, an approach that can decrease the leakage-factor by optimizing the GRAPPA technique for slice direction, was reported by Cauley et al.^24^ This technique could decrease the leakage factor, while maintaining image quality with high MBf.

**Fig 7.**
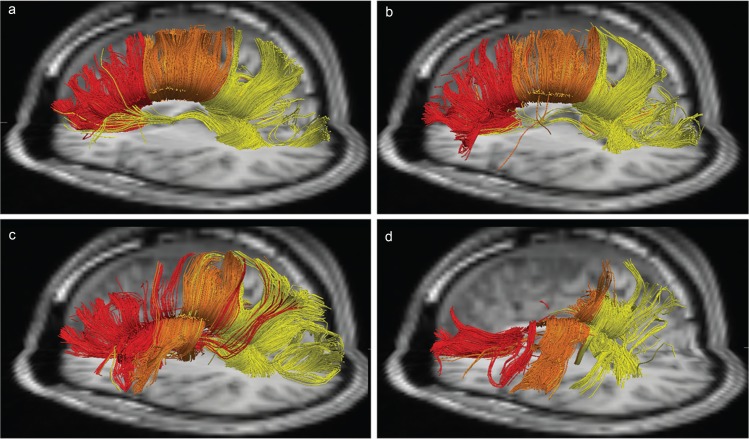
Diffusion tensor tractography (DTT) of corpus callosum (CC) on T_1_-weighted imaging under the following conditions: SB-EPI (**a**), MBf2 (**b**), MBf3 (**c**), and MBf4 (**d**). As MBf increased, DTT of CC were less accurately imaged than with SB-EPI.

In addition, we confirmed another fiber bundle, which is the corpus callosum. The result image is shown in Fig. 7. The imaging capacity tended to be similar to the DTT of the pyramidal tracts. It is likely that MB-EPI is useful for the DTT of pyramidal tracts as well as that of other fiber tracts. We would like to consider about other fiber bundles in further research.

This study demonstrated that MB-EPI using 1.5T MRI and a 12 channel coil, which are widely used in clinical practice, is useful for DTI and DTT. DTT is used for imaging nerve fibers, but it is difficult to image crossing and adjoining fibers within an individual voxel by DTT.^25^ To resolve this issue, some methods (e.g., diffusion spectrum imaging, q-ball imaging, and high-angular resolution diffusion imaging) have been proposed.^9,26,27^ We plan to apply MB-EPI with these techniques, in an attempt to obtain high precision tractography images using a short imaging time.

## Conclusion

We have demonstrated that MB-EPI, using MBf2 is a useful tool for DTI and DTT employing 1.5T MRI. The technique may facilitate the use of DTI and DTT in clinical scenarios.
